# Antioxidant and In Vitro Preliminary Anti-Inflammatory Activity of *Castanea sativa* (Italian Cultivar “Marrone di Roccadaspide” PGI) Burs, Leaves, and Chestnuts Extracts and Their Metabolite Profiles by LC-ESI/LTQOrbitrap/MS/MS

**DOI:** 10.3390/antiox10020278

**Published:** 2021-02-11

**Authors:** Antonietta Cerulli, Assunta Napolitano, Jan Hošek, Milena Masullo, Cosimo Pizza, Sonia Piacente

**Affiliations:** 1 Dipartimento di Farmacia, Università degli Studi di Salerno, via Giovanni Paolo II n. 132, I-84084 Fisciano, SA, Italy; acerulli@unisa.it (A.C.); anapoli@unisa.it (A.N.); mmasullo@unisa.it (M.M.); pizza@unisa.it (C.P.); 2 Department of Pharmacology and Toxicology, Veterinary Research Institute, Hudcova 296/70, 621 00 Brno, Czech Republic; hosek.jan@vri.cz

**Keywords:** “Marrone di Roccadaspide” PGI, leaves, burs, chestnuts, antioxidant activity, anti-inflammatory activity, LC-ESI/LTQOrbitrap/MS/MS analysis, phenolic compounds, polar lipids

## Abstract

The Italian “Marrone di Roccadaspide” (*Castanea sativa*), a labeled Protected Geographical Indication (PGI) product, represents an important economic resource for the Italian market. With the aim to give an interesting opportunity to use chestnuts by-products for the development of nutraceutical and/or cosmetic formulations, the investigation of burs and leaves along with chestnuts of *C. sativa*, cultivar “Marrone di Roccadaspide”, has been performed. The phenolic, tannin, and flavonoid content of the MeOH extracts of “Marrone di Roccadaspide” burs, leaves, and chestnuts as well as their antioxidant activity by spectrophotometric methods (1,1-diphenyl-2-picrylhydrazyl (DPPH), Trolox Equivalent Antioxidant Capacity (TEAC), and Ferric Reducing Antioxidant Power (FRAP) have been evaluated. Furthermore, a cell-based antioxidant in vitro test along with in vitro assays for the evaluation of the ability to reduce nuclear factor-kappa B (NF-κB) activation and nitric oxide (NO) production have been carried out. In order to identify the secondary metabolites responsible for the high phenolic content and the strong antioxidant activity shown by leaves and burs extracts, and to highlight the differences between their chemical composition, the analysis of the metabolite profile of the MeOH extracts obtained from both by-products and chestnuts by liquid chromatography coupled to electrospray ionization and multiple-stage linear ion-trap and Orbitrap high-resolution mass spectrometry (LC-(-)ESI/LTQOrbitrap/MS/MS) has been performed. LC-MS analysis allowed the identification of different classes of specialized metabolites including hydrolyzable tannins, flavonoids, ellagic acid and phenol glucoside derivatives, and triterpenoids as well as polar lipids. Our results show how the antioxidant activity of the extracts can be correlated to their high tannins and flavonoids content while polar lipids occurring in the MeOH extract of the leaves could contribute to determining its higher anti-inflammatory activity.

## 1. Introduction

*Castanea sativa* Mill. is a deciduous tree of the Fagaceae family that can be found in south Europe and Asia (China) [1]. Chestnut is composed of the fruit, pericarp (outer shell), integument (inner shell), and bur that surrounds the edible nuts. These fruits, consumed raw or after cooking, are a highly appreciated seasonal (autumn) nut in the Mediterranean countries [2,3]. Italy, with France, Spain, and Portugal, is one of the largest European producers of sweet chestnuts (*Castanea sativa* Mill., Fagaceae family). Campania region provides 50% of the chestnut national crops and ‘Marrone di Roccadaspide’, a labeled PGI (Protected Geographical Indication) product, represents an important economic resource for the Campania market [4].

During industrial processes, only chestnuts are used for food preparations such as marron glacé, purées, and chestnut flour, which finds increasing application as an ingredient in gluten-free diets, while shells, leaves, as well as burs may be considered by-products of the food industry [5,6]. In the literature, there are different studies that highlight the presence of bioactive compounds in the waste of *Castanea sativa* showing health benefits. Among them, antioxidant activity is the most important [7,8,9]. Oxidative stress is generated when the balance between reactive species formation and their elimination is disrupted. Under this biological condition, there is an overproduction of reactive oxygen species (ROS) such as superoxide (O_2_^−^), hydroxyl radical (OH), and hydrogen peroxide (H_2_O_2_), as well as of reactive nitrogen species (RNS) such as nitric oxide (NO) and peroxynitrite (ONOO^−^), overall leading to several mitochondrial and cellular damages. Oxidative stress also plays a critical role in the triggering and progression of inflammation through the overproduction of ROS and RNS. The overproduction of pro-inflammatory factors including nitric oxide (NO), prostaglandin E2 (PGE2), cyclooxygenase-2 (COX-2), and cytokines such as tumor necrosis factor (TNF-α) and interleukin-6 (IL-6) increases the inflammatory response. Furthermore, they activate a transcription factor such as nuclear factor-kappa B (NF-kB), which accelerates pathogenic inflammation and the development of chronic diseases [10].

Our previous investigation on “Marrone di Roccadaspide” shell extract, rich in hydrolyzable and condensed tannins as well as flavonoids and triterpenoids, showed its capability to modulate the pro-inflammatory transcriptional factor NF-κB after lipopolysaccharide (LPS) stimulation and to exert a good antioxidant activity by a cell-based in vitro test [11]. Moreover, phenolic compounds isolated from the MeOH extract of “Marrone di Roccadaspide” leaves were reported to prevent UV-induced/cell damage [12].

Thereby, supported by these premises, and with the aim to give an added value to burs and leaves by-products as well as to chestnuts themselves as a source of bioactive phytochemicals, herein, the MeOH extracts of “Marrone di Roccadaspide” burs, leaves, and chestnuts have been investigated for the total phenolic and tannin content determined by the Folin–Ciocalteu method, for the total flavonoid content and for the antioxidant activity, tested by the 1,1-diphenyl-2-picrylhydrazyl (DPPH) Radical Scavenging test, Trolox Equivalent Antioxidant Capacity (TEAC) assay, and the Ferric Reducing Antioxidant Power (FRAP) test. Furthermore, a cell-based antioxidant in vitro test along with in vitro assays for NF-κB determination and NO production were performed.

To correlate the antioxidant and the anti-inflammatory activities to the chemical composition, the metabolite analysis of MeOH extracts of burs, leaves, and chestnuts by an approach based on liquid chromatography coupled to electrospray ionization and multiple-stage linear ion-trap and Orbitrap high-resolution mass spectrometry (LC-ESI/LTQOrbitrap/MS/MS), in negative ion mode has been carried out. In this way, the identification of specialized metabolites belonging to the classes of hydrolyzable tannins (galloyl glucose derivatives and ellagitannins), flavonoid glycosides, ellagic acid and phenolic derivatives as well as triterpenoids was accomplished. Furthermore, a wide range of polar lipids (phospholipids, sphingolipids, and glycolipids) has been identified. Moreover, the content of the main phenolic constituents has been determined by LC-ESI/QTrap/MS/MS.

## 2. Materials and Methods

### 2.1. Chemicals and Reagents

MeOH and water for HPLC were purchased from VWR (Milan, Italy). Acetonitrile, HCOOH, and water for LC-MS analysis were bought from Merck (Merck KGaA, Darmstadt, Germany) MeOH-d4 (99.95%), Folin–Ciocalteu, DPPH, polyvinylpolypyrrolidone (PVPP), 6-hydroxy-2,5,7,8- tetramethylchroman-2-carboxylic acid (Trolox), NaNO_2_, AlCl_3_, NaOH, diammonium 2,2′-azinobis(3-ethylbenzothiazoline-6-sulfonate) (ABTS), Trolox, ellagic acid, quercetin, vitamin C, 2,3,5-Triphenyltetrazolium chloride (TPTZ), HCl FeCl_3_ × 6 H_2_O, FeSO_4_, LPS, prednisone, pyocyanin (PCN), 2′-7′-dichlorodihydrofluorescein diacetate (DCFH2-DA), and L-1-p-Tosylamino-2-phenylethyl chloromethyl ketone (TPCK) reagents were purchased by Sigma-Aldrich (Milan, Italy).

WST-1 reagent was purchased from Roche (Basel, Switzerland). QUANTI-Blue was bought from InvivoGen (San Diego, CA, USA), Methylthiazolyldiphenyl-tetrazolium bromide (MTT) was purchased by Sigma-Aldrich (Milan, Italy).

The THP-1 human monocytic leukemia cell line was purchased from the European Collection of Cell Cultures (Salisbury, UK). THP-1-XBlue-MD2-CD14 cell line (Invivogen; San Diego, CA, USA) has been derived from THP-1. The cultured murine monocyte macrophage cell line J774.A1 was purchased from the American Type Tissue Culture Collection (Rockville, MD, USA).

### 2.2. Plant Material

Burs, leaves, and chestnuts of *C. sativa* Mill., cultivar “Marrone di Roccadaspide” PGI, were provided as a kind gift by Azienda Agricola Vona Fiorenza, GPS coordinates N: 40°25′23.988″, S: 15°11′28.247″ (Roccadaspide, Salerno, Italy) in October 2018.

Burs and shells were immediately separated from the fruits by hand to carry out the analysis. A voucher specimen for each part was identified by Prof. V. De Feo, Department of Pharmacy, University of Salerno, and deposited at this Institution.

### 2.3. Extraction of “Marrone di Roccadaspide” Leaves, Burs, and Fruits

The leaves of *C. sativa* (40.0 g) were dried and extracted at room temperature using solvents of increasing polarity such as petroleum ether (0.6 L for 3 days, three times), chloroform (0.6 L for 3 days, three times), and methanol (0.6 L for 3 days, three times). After filtration and evaporation of the solvent to dryness *in vacuo*, 4.75 g of crude MeOH extract were obtained.

The burs of *C. sativa* (34.3 g) were dried and extracted at room temperature using solvents of increasing polarity such as petroleum ether (0.44 L for 3 days, two times), chloroform (0.44 L for 3 days, two times), and methanol (0.44 L for 3 days, three times). After filtration and evaporation of the solvent to dryness *in vacuo*, 3.98 g of crude MeOH extract were obtained.

The fruits of *C. sativa* (100 g) were dried and extracted at room temperature using solvents of increasing polarity such as petroleum ether (0.4 L for 3 days, two times), chloroform (0.4 L for 3 days, three times), and methanol (0.4 L for 3 days, three times). After filtration and evaporation of the solvent to dryness *in vacuo*, 13.26 g of crude MeOH extract were obtained. The obtained MeOH extract was subjected to *n*-butanol/water partition to remove free sugars; the dried butanolic extract was resolubilized in MeOH and used for LC-MS analyses.

### 2.4. Determination of Total Phenolic, Total Tannin, and Total Flavonoid Content

To determine the total phenolic content of the extracts, the Folin–Ciocalteu assay was employed, as reported by Masullo et al. [13] As a standard reference, gallic acid was used. For the calibration curve, 5, 10, 20, 30, 40, and 50 μg/mL solutions of gallic acid were prepared and submitted to the assay following the same procedure used for the extracts. For gallic acid, the calibration equation was y = 0.0027x + 0.1242 (R^2^ = 0.995). All the experiments were performed in triplicate, and results expressed as mean of gallic acid equivalents (GAEmg/g dried extract). 

Total tannin content of extracts was determined via polyvinylpolypyrrolidone (PVPP) [14]. Each extract (1 mL) was treated with PVPP (200 mg), mixed with distilled water (2 mL), and vortexed for 10 min. The reaction mass was kept at 40 °C for 2 h and after centrifugation, the supernatant was collected and the same procedure described for the determination of the total phenolic content was carried out. The supernatant contains the phenolic constituents other than tannins, precipitated with the addition of PVPP. From the above results, the Total Tannin Content of the various extracts was calculated as follows:

Tannins (%) = Total Phenolics (%) − Non-Tannin Phenolics (%).(1)

To determine the flavonoid content, a known volume of extract (1 mg/mL) was placed in a 10 mL volumetric flask. Distilled water (5 mL) and a solution of NaNO_2_ (1:20) (0.3 mL) were added. A solution of AlCl_3_ (1:10) (3 mL) was added 5 min later. After 6 min, NaOH (1 M) (2 mL) and distilled water up to 10 mL were added. The solution was mixed, and the absorbance was measured against a blank at 510 nm. Rutin was used as standard for a calibration curve (y = 0.0101x + 0.348, R^2^ = 0.999) [15].

### 2.5. Determination of DPPH Radical Scavenging Activity, Antioxidant Activity by TEAC Assay, and Ferric Reducing Antioxidant Power (FRAP)

The DPPH and TEAC assays have been determined as previously reported [13]. In detail, the DPPH• concentration in the reaction medium was calculated from a calibration curve (range = 5–50 μg/mL) using vitamin C as positive control and analyzed by linear regression (y = 0.213x + 40.77, R^2^= 0.995). In the TEAC assay (range= 0.25–1.00 mg/mL), the antioxidant activities of analyzed extracts were expressed as TEAC values in comparison with the TEAC activity of quercetin 3-*O*-glucopyranoside, which was used as a reference compound. The TEAC value is defined as the concentration of a standard Trolox solution with the same antioxidant capacity as 1 mg/mL of the tested extract.

The FRAP assay was performed as reported by Zamuz et al. [16]. The FRAP reagent was freshly prepared with 25 mL of 300 mmol/L acetate buffer (pH 3.6), 2.5 mL of a 10 mM solution of TPTZ in 40 mmol/L HCl, and 2.5 mL of 20 mM FeCl_3_ × 6 H_2_O in distilled water. This solution was used as a blank, and a solution of 1 mM ascorbic acid was used for calibration. Then, 3 mL of the FRAP reagent were mixed with 100 μL of extract, and the absorbance was monitored at 593 nm. FeSO_4_ aqueous solutions were used for calibration.

### 2.6. Biological Assays

#### 2.6.1. Determination of Cytotoxicity by WST-1 Assay

The cytotoxicity of the MeOH extracts and their ability to inhibit proliferation (WST-1 assay) were evaluated. THP-1 cells (floating monocytes, 500,000 cells/mL) were incubated in 100 µL of a serum-free RPMI 1640 medium and seeded into 96-well plates in triplicate at 37 °C. Increasing concentrations of the test extracts (2, 5, 10, and 50 µg/mL) dissolved in DMSO (the final concentration of DMSO was 0.1% (*v*/*v*) in the cultivation medium) were added. After 24 h from treatment with extracts, measurements were carried out. Viability was evaluated by the cell proliferation reagent WST-1 (Roche, Basel, Switzerland) according to the manufacturer’s manual. The amount of formazan created (which correlates to the number of metabolically active cells in the culture) was calculated as a percentage of the control cells, which were treated only with DMSO and were assigned as 100% [17,18].

#### 2.6.2. Determination of Intracellular ROS Scavenging Activity

To highlight the possible pro/anti-oxidative activity of the different extracts, a dichlorofluorescein method was used. THP1-XBlueTM-MD2-CD14 cells (Invivogen, San Diego, CA, USA) were seeded in a serum-free medium (500,000 cells/100 µL/well) into a black 96-well plate and were incubated for 2 h. After incubation, the solutions of test extracts were added at the final concentration of 5 µg/mL, and the standard antioxidant quercetin was added at the final concentration of 5 µM. After 60 min of the treatment of the cells with the compounds, pyocyanin was added in a final concentration of 100 µM in each well (except in the group of negative control), and the cells were incubated for 30 min. At the end, DCFH2-DA (5 µg/mL) was added into the cell medium of all the wells, and the cells were incubated for the next 30 min. Finally, the intracellular fluorescence of the dichlorofluorescein product was measured by a Fluostar Omega MicroplateReader (BMG Labtech) using the λ (ex./em.) = 480/530 nm [11].

#### 2.6.3. Determination of Intracellular NF-κB Activity

THP1-XBlue-MD2-CD14 cells express an NF-kB/activator protein 1 (AP-1)-inducible Secreted Embryonic Alkaline Phosphatase (SEAP) reporter gene. The activation of NF-κB and AP-1 leads to subsequently the production of SEAP; to measure SEAP protein, we used QUANTI-Blue (Invivo Gen, San Diego, CA, USA), which is a medium that shows a different range of color from purple to blue, depending on the amount of SEAP produced. The cells were seeded (50,000 cells/100 µL/well) in a 96-well plate and successively were incubated for 2 h. After the incubation time, the cells were pretreated for 1 h with the test extracts at the final concentration of 5 µg/mL and prednisone (reference) at the final concentration of 1 µM. After the pre-incubation, 2 µL of LPS (1 µg/mL) was added to each well to activate the NF-kB pathway. Measurements were taken about 24 h after treatment, and the activity of NF-kB was determined spectrophotometrically by a Fluostar Omega Microplate Reader (BMG Labtech) [11].

#### 2.6.4. Determination of Cytotoxicity by MTT Assay

In order to evaluate the effect of extracts on cell viability, cells from the J774.A1 cell line (3.5 × 10^4^/well) were seeded on a 96-well multiwell and allowed to adhere for 4 h at 37 °C in a 5% CO_2_ atmosphere. Thereafter, the medium was replaced with fresh medium and serial dilutions of extracts (2, 5, 10, and 50 µg/mL) were added for 72 h. Cell viability was assessed through MTT assay as previously reported [19]. Briefly, 25 µL of MTT (5 mg/mL) were added, and cells were incubated for additional 3 h. Thereafter, cells were lysed and the dark blue crystals were solubilized with 100 µL of a solution containing 50% (v:v) N,N-dimethylformamide, 20% (w:v) SDS with an adjusted pH of 4.5. The optical density (OD) of each well was measured with a microplate spectrophotometer (Titertek Multiskan MCC/340) equipped with a 620 nm filter. Macrophage cell viability in response to treatment with extracts was calculated as: % dead cells= 100 × [(OD treated/OD control) × 100] [19].

#### 2.6.5. Analysis of Nitrite Production

J774.A1 cells were maintained in

Dulbecco’s Modified Eagle Medium (DMEM) supplemented with NaHCO_3_ (42 mM), Hepes (25 mM), penicillin (100 units/mL), streptomycin (100 units/mL), glutamine (2 mM), and fetal calf serum (FCS, 10%) at 37 °C in a 95% air and 5% CO2 atmosphere. Macrophages J774A.1 were seeded in 96-well multiwell plates (5.0 × 10^4^/well) and allowed to adhere for 2 h. Thereafter, the medium was replaced with fresh medium, and cells were pretreated with extracts (5 μg/mL) and TPCK (reference) at the final concentration of 5 µM. After 1 h, LPS (1 μg/mL) was added, and cells were incubated for 24 h. NO production was measured as nitrite (NO^2−^, μM) released in the medium of LPS-activated J774.A1 macrophages, index of nitric oxide (NO) produced and released by cells, in the culture medium 24 h after LPS stimulation, as previously reported. NO^2−^ amounts were measured by Griess reaction. Briefly, 100 μL of cell culture medium were mixed with 100 μL of Griess reagent equal volumes of 1% (w:v) sulfanilamide in 5% (v:v) phosphoric acid and 0.1% (w:v) naphtylethylenediamine-HCl and incubated at room temperature for 10 min; then, the absorbance was measured at 550 nm in a microplate reader Titertek (Dasit, Cornaredo, Milan, Italy) [20].

### 2.7. LC-ESI/LTQOrbitrap/MS/MS Analysis

Qualitative LC-MS was performed by using an Accela HPLC system equipped with an RP C18 Atlantis T3 column (150 mm × 2.1 mm i.d., 5 μm) and working at a flow rate of 0.2 mL/min, coupled to a LTQ-Orbitrap XL mass spectrometer (Thermo Fisher Scientific, San Jose, CA, USA) operating in the negative ion mode.

The Orbitrap mass analyzer was calibrated according to the manufacturer’s directions using a mixture of caffeine, methionine-arginine phenylalanine-alanine-acetate (MRFA), sodium dodecyl sulfate, sodium taurocholate, and Ultramark 1621. Data were collected and analyzed using the software provided by the manufacturer [21].

Linear gradient elution was carried out by using water as eluent A and acetonitrile as eluent B, both with 0.1% formic acid. MeOH extracts of burs, leaves, and chestnuts were analyzed by using a linear gradient increasing from 10 to 35% B in 20 min, held to 35% B for 5 min, increased to 55% B in 12 min, to 100% B in 20 min, and held to 100% B for 8 min. The MeOH extract of chestnuts was further analyzed by the following linear gradient: 10 min at 35% B, increased to 55% B in 2 min, to 60% B in 5 min, held to 60% B for 15 min, increased to 65% B in 5 min, to 75% B in 3 min, to 100% B in 5 min, and held to 100% B for 10 min.

The autosampler was set to inject 6 μL of each extract (0.5 mg/mL). The scan was collected in the Orbitrap at a resolution of 30,000 in a *m*/*z* range of 220–1500 Da. The *m*/*z* of each identified compound was calculated to 4 decimal places and measured with a mass accuracy < 2 ppm. The (-)ESI parameter settings were: capillary temperature at 280 °C, sheath gas flow at 15 (arbitrary units), auxiliary gas flow at 5 (arbitrary units), source voltage at 3.5 kV, capillary voltage at −48 V, and tube lens offset at −176.47 V. In LC-(-)ESI/HRMS experiments, the Total Ion Current (TIC) profile was produced by monitoring the intensity of all the ions produced and acquired in every scan during the chromatographic run. In order to get structural information, data-dependent experiments were performed by acquiring MS/MS spectra of the first and the second most intense ions produced during the HRMS scan event. A normalized collision energy at 30%, a minimum signal threshold at 250, and an isolation width at 2.0 were used.

### 2.8. LC-ESI/QTrap/MS/MS Analysis

Quantitative analysis was performed on a LC-ESI/QTrap/MS system, working in Multiple Reaction Monitoring (MRM) mode, and using a Kinetex EVO 1.7 µm RP C18 column (Phenomenex, 100 × 2.1 mm) kept at 30 °C, a flow rate of 0.23 mL/min, and a mobile phase consisting of a combination of A (0.1% HCOOH in water, *v*/*v*) and B (0.1% HCOOH in acetonitrile, *v*/*v*). A linear gradient from 10 to 35% B in 3.4 min, held to 35% B for 45 s, from 35 to 55% B in 2.04 min, from 55 to 100% B in 3.4 min, held to 100% B for 2.36 min, and from 100 to 10% in 17 s, was used. The autosampler was set to inject 4 µL of extract (500 ng/µL) [11].

### 2.9. Calibration and Quantification

Stock solutions (1 mg/mL) of isolated compounds used as external standards (ES) were prepared by dissolving each compound in a solution of acetonitrile/water (70:30 v/v). Each stock solution was diluted with appropriate amounts of acetonitrile to obtain nine solutions of different ES concentration (0.01, 0.05, 0.5, 1.0, 2.5, 5, 10, 25, and 35 ng/µL). To each ES solution as well as to the burs and leaves of MeOH extracts, an appropriate amount of internal standard (IS; resveratrol) was added to yield a final concentration of 1 ng/µL. Calibration curves were constructed by injecting 2 µl of each standard solution at each concentration level in triplicate. The ratios of the peak areas of the ES to those of the IS were calculated and plotted against the corresponding concentrations of the standard compounds using weighted linear regression to generate standard curves. Linear regression analysis was performed using the Analyst 1.6.2 Software provided by the manufacturer (AB Sciex).

In order to validate the LC-ESI/QTrap/MS/MS method, precision (at nine concentrations for each compound), specificity, linear range, limit of detection (LOD), and limit of quantification (LOQ) were evaluated. The limit of detection (LOD) and the limit of quantification (LOQ) for each target standard compound were determined, under the optimized conditions, by the serial dilution of a standard solution until the signal-to-noise ratios (S/Ns) were 3:1 and 10:1, respectively. The LOD for each analyte varied from 0.006 to 0.066 µg/µL and LOQ from 0.021 to 0.22 µg/µL [22].

### 2.10. Statistical Analysis

Statistical analyses were carried out using GraphPad Prism 7.0 software (San Diego, CA, USA). The data were graphed as means SEM. Comparisons between groups were made using a one-way analysis of variance test, followed by the Bonferroni’s multiple comparisons test.

## 3. Results

### 3.1. Evaluation of Total Phenolic, Tannin, and Flavonoid Content of C. sativa “Marrone di Roccadaspide” Burs, Leaves, and Chestnuts by Spectrophotometric Methods

The results of phenolic content evaluation by the Folin-Ciocalteu method indicated a high phenolic content in both burs (580.44) and leaves (298.96) extracts, which were expressed as milligrams of gallic acid equivalents (GAE) for gram of extract, but there was no phenolic occurrence in chestnuts extract. Regarding the estimation of tannin and flavonoid content, in agreement with the results of the determination of phenolic content, burs showed the highest tannin (276.44) and flavonoid content (87.19) (Table 1). The chestnuts extract showed total tannin and flavonoid contents so low to not be detectable.

### 3.2. Evaluation of the Antioxidant Activity of C. sativa “Marrone di Roccadaspide” Burs, Leaves and Chestnuts by Spectrophotometric Methods

Leaves and burs showed a high antioxidant activity evaluated by spectrophotometric methods, displaying a significant concentration-dependent free-radical scavenging activity by DPPH (EC_50_ = 4.21 µg/mL burs and EC_50_ = 3.06 µg/mL leaves), which was comparable to vitamin C (EC_50_ = 4.00 µg/mL) used as a reference compound; antioxidant activity was observed also with TEAC assay (3.03 mg/mL burs and 3.01 mg/mL leaves) (Table 2). The antioxidant activity was also evaluated by FRAP assay (2.96 mmol/g burs and 1.48 mmol/g leaves) (Table 2).

### 3.3. In Vitro Cytotoxicity Analysis

The effect of MeOH extracts of “Marrone di Roccadaspide” leaves, burs, and chestnuts on the viability of cells of THP-1 and THP-1-XBlue-MD2-CD14 cell lines was assessed using a WST-1 assay to detect metabolically active cells. The aim of the assay was to determine the concentration of each compound that would be safe, would not influence the viability of cells, and could be used for subsequent in vitro experiments. The amount of formazan formed by reduction of the tetrazolium salt WST-1 corresponds directly to the number of viable cells with active mitochondrial reductases. All cell cultures showed similar results. A clearly non-toxic concentration of the test extract was chosen for subsequent studies on cell cultures (5 µg/mL).

### 3.4. Evaluation of In Vitro Antioxidant Activity

The antioxidant effects of MeOH extracts of “Marrone di Roccadaspide” burs, leaves, as well as chestnuts, by the specific fluorogenic probe DCFH-DA, were assayed. DCFH-DA, a lipophilic and non-fluorescent compound, passes through a plasma membrane and de-esterifies to form the hydrophilic phenol DCFH_2_ (dihydrodichlorofluorescein), which can be oxidized to fluorescent DCF (2’-7’-dichlorofluorescein) by a process that is usually thought to involve ROS. LPS quickly increases ROS in human monocytic leukemia cells during a 90 min incubation, and this probably led to the peroxidation of lipids in the membrane. A previous pre-incubation with the above-mentioned extracts significantly reduced the oxidative stress (*p* < 0.001).

**Figure 1:**
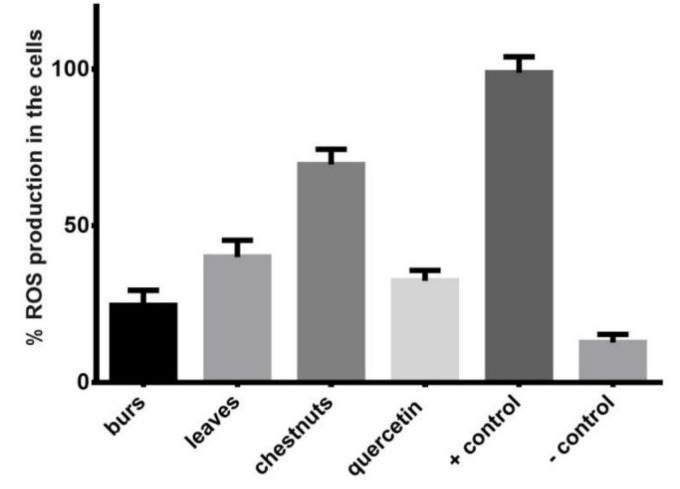
Antioxidant activity of the MeOH extract of “Marrone di Roccadaspide” Protected Geograph ical Indication (PGI) leaves, burs, and chestnuts by in vitro 2-7dichlorodihydrofluorescein diacetate (DCFH2-DA) cell-based assay. Significant difference in comparison to vehicle only treated cells (*p* < 0.001). + control = cells + LPS; − control = only cells.

In detail, at 5 µg/mL, the leaves and burs extracts reduced intracellular levels of ROS, which were produced after stimulation of the THP-1-XBlue-MD2-CD14 cells with PCN, in a percentage of 58.84% ± 2.86% and 74.30% ± 2.96%, respectively, showing an antioxidant activity similar or higher than quercetin (66.41% ± 2.58%), which was used as a reference compound (Figure 1).

### 3.5. Effects of Extracts on NF-κB Activation

**Figure 2:**
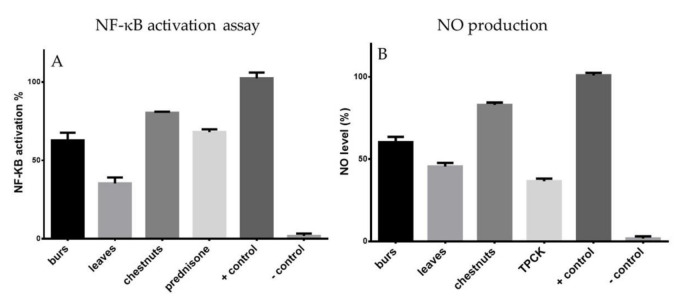
Anti-inflammatory activity of the MeOH extract of *C. sativa* (Marrone di Roccadaspide) leaves, burs, and chestnuts by in vitro nuclear factor-kappa B (NF-κB) activation assay (**A**) and nitric oxide (NO) production assay (**B**). Results are expressed as mean ± SE for three independent experiments. Significant difference in comparison to vehicle only treated cells (*p* < 0.001). + control = cells + LPS; -control = only cells.

The ability of MeOH extracts of “Marrone di Roccadaspide” leaves, burs, and chestnuts to modulate the activity of the important pro-inflammatory transcriptional factor NF-κB after LPS stimulation was investigated. LPS significantly (*p* < 0.001) increased the activity of NF-κB in THP1-XBlue-MD2-CD14 cells. After stimulation with LPS, tested extracts, as well as the well-known anti-inflammatory drug prednisone, reduced the NF-κB activity. The MeOH extracts of leaves and burs, at 5 µg/mL concentration, reduced NF-κB activation to 35.31% ± 1.69% and 62.73% ± 1.59%, respectively, showing an activity comparable to that exerted by prednisone (63.01% ± 2.02%), used as reference compound (Figure 2), at 1 µM.

### 3.6. Effects of Extracts on NO Production

Preliminarily, the effect of MeOH extracts on the viability of J774.A1 cells was performed using a MTT assay [19] to select a non-toxic concentration (5 µg/mL) of the test extract for subsequent studies on cell cultures. To assess if MeOH extracts of “Marrone di Roccadaspide” PGI leaves, burs, and chestnuts could interfere in LPS-induced J774.A1 macrophages activation, the release of nitrite, a stable end product of NO production in cellular medium of the LPS-activated murine macrophages J774.A1 incubated with extracts was measured. After stimulation with LPS, extracts added to J774 macrophages, determined a significant (*p* < 0.001) inhibition of nitrite production in cell medium to 60.23% ± 0.97%, 45.55% ± 1.04% and 82.96% ± 1.09% for burs, leaves, and chestnuts, respectively. The well-known anti-inflammatory TPCK was used as reference compound (36.59% ± 1.09%) (Figure 2).

### 3.7. LC-MS Analysis of C. sativa Leaves, Burs, and Chestnuts

#### 3.7.1. LC-MS Analysis of Specialized Metabolites Occurring in MeOH Extract of *C. sativa* Leaves, Burs, and Chestnuts

An analytical approach based on LC-(-)ESI/LTQOrbitrap/MS/MS was carried out with the dual aim to identify the specialized metabolites occurring in MeOH extracts of “Marrone di Roccadaspide” leaves and burs, which are likely responsible for their antioxidant and anti-inflammatory activities, and to highlight the metabolite differences between them and the chestnut extract. According to their accurate mass, characteristic fragmentation pattern, retention time, as well as literature data, the identity of most of the peaks was putatively attributed. In particular, five main classes of specialized metabolites, i.e., hydrolyzable tannins as ellagitannins and galloyl glucose derivatives, flavonoids, triterpenoids, and derivatives of phenol glucoside and ellagic acid, could be detected both in burs and in leaves, but not in chestnuts, only showing the presence of ellagic acid (**60**) (Table 3 and Table 4).

The hydrolyzable tannin class was one of the most abundant classes detected both in burs and in leaves. Chemically, the hydrolyzable tannin class is composed of galloyl glucose derivatives, consisting of metabolites in which up to five gallic acid units can be esterified directly to D-glucose, and ellagitannins (ETs). These latter are derived from pentagalloyl glucose by the oxidative coupling of two (or more) neighboring galloyl groups. The hexahydroxydiphenoyl (HHDP) group formed is a basic structure for the majority of the ET monomers. The HHDP group may be coupled with another galloyl group to form the nonahydroxytriphenoyl (NHTP) group characteristic to C-glycosidic ETs with an open glucose core [11].

Numerous compounds (**4**–**6**, **12**–**14**, **17**, **19**–**20**, **22**–**24**, **26**, **28**–**30**, **33**, **35**, **40**, **43**–**47**, **49**–**50**, **57**, and **68**) occurring both in burs and in leaves showed mass spectrometric data supporting their belonging to different ET sub-classes (Table 3). According to the typical behavior of ETs [23], in most cases, negative LC-HRMS spectra of monomeric ETs showed, in addition to negative ions corresponding to the [M-H]^−^ deprotonated molecule, [M-2H]^2−^ doubly charged molecular ions, allowing the determination of molecular formula (Table 3). Moreover, careful study of the fragmentation pattern produced from these metabolites in tandem mass experiments allowed putatively assigning their chemical structure, considering that the fragmentation pathway was characterized by the presence of highly diagnostic product ions, which originated by typical neutral losses such as those of ellagic acid (302 Da), galloyl (152 Da), galloyl-glucose (332 Da), HHDP-glucose (482 Da), and galloyl-HHDP-glucose (634 Da) moiety. Furthermore, considering that the structural features of HHDP- and NHTP-glucose derivatives determine the molecular weights of HHDP and NHTP esters with the same number of galloyl units, such as galloyl-HHDP-glucose (molecular weight 634 Da) and NHTP-glucose (molecular weight 632 Da), which differ by 2 Da, it was possible to discriminate between these species and to hypothesize, as in the case of compounds **17** and **29** (Table 3), that the first was a NHTP-HHDP-glucose, as further confirmed by the analysis of the whole fragmentation pattern [11].

The accurate analysis of *m*/*z* values of peaks **3**, **8**, **10**–**11**, **25**, **34**, **36**, and **58** along with their MS/MS spectra, which were mainly characterized by the occurrence of product ions formed by the neutral loss of one or more galloyl groups (152 mass units) and/or gallic acid (170 mass units), allowed identifying them as hydrolyzable tannins, too, but in this case as galloyl glucose derivatives (Table 3). In particular, digalloyl- and trigalloyl-glucose along with tetragalloyl-glucose isomers could be detected [12].

The second main class of secondary metabolites detectable in chestnut burs and leaves consisted of flavonoids, in particular of glycosilated flavonols such as quercetin (**48**, **54**–**55**, **62**, **63**, **72, 91**), isorhamnetin (**51**, **61**, **66**, **78**, **83**), and kaempferol derivatives (**52**, **59**, **75**–**77**, **79**, **81**, **87**, **99**, **106**, **108**–**110**, **113**–**116**) (Table 3). These metabolites yielded fragmentation patterns in which the base peak was produced by neutral loss of the sugar unit, which was mainly represented by hexose (neutral loss of 162 Da) and deoxyhexose (neutral loss of 146 Da) moiety. Moreover, some metabolites corresponding to flavonoid glycosides acylated with aliphatic (acetyl) and aromatic (galloyl, coumaroyl, and caffeoyl) moieties could be identified by considering both their molecular formula and fragmentation pattern, which was characterized by the occurrence of product ions originated by neutral loss of 60, 152, 146, and 162 Da, respectively (Table 3) [8,13,24,25,26].

**Figure 3:**
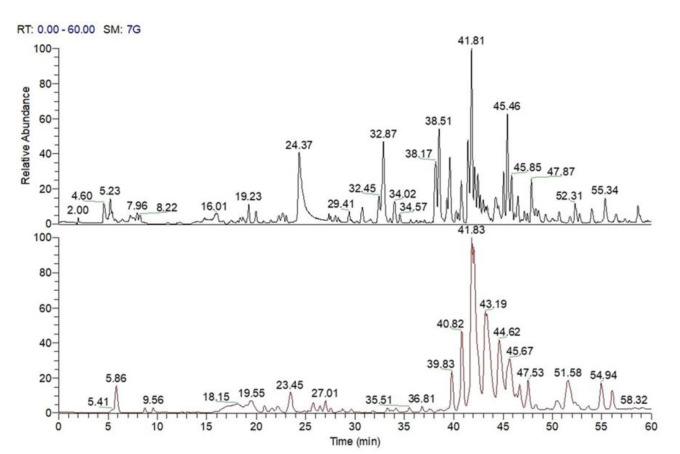
Liquid chromatography coupled to electrospray ionization and multiple-stage linear ion-trap and Orbitrap high-resolution mass spectrometry (LC-ESI/LTQOrbitrap/MS) profiles (negative ion mode) of the MeOH extract of “Marrone di Roccadaspide” PGI burs (black) and leaves (red).

The analysis of LC-HRMS spectra along with tandem mass fragmentation pattern allowed assigning peaks **7**, **18**, **21**, **27**, **31**–**32**, **38**, **41**, and **56** to phenol glucoside derivatives, which is a metabolite class already described in *C. sativa* (Table 3), and peaks **42**, **53**, **60**, **65**, **71**, **73–74**, **84**–**85**, **89**, and **112** to ellagic acid derivatives, on the basis of their mass spectrometric behavior (Table 3). Finally, in agreement with previous reports [11,27], peaks **69**, **80**, **82**, **88**, **94**, **96**, and **119** could be putatively identified as triterpenoid compounds (Figure 3; Table 3).

#### 3.7.2. LC-MS Qualitative Analysis of Polar Lipids in MeOH Extract of *C. sativa* Burs, Leaves, and Chestnuts

**Figure 4:**
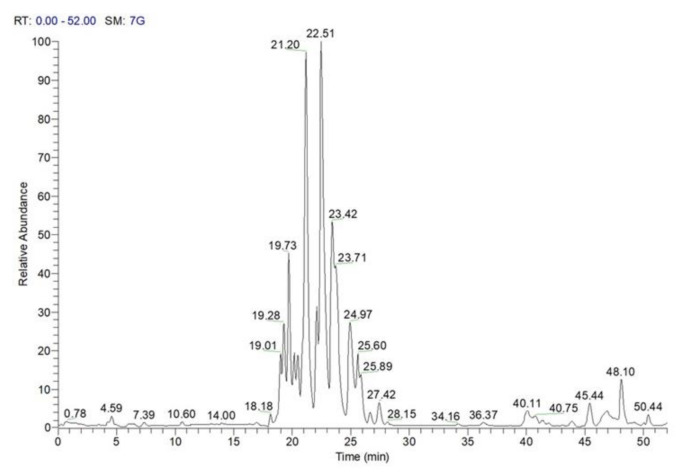
LC-ESI/LTQOrbitrap/MS profile (negative ion mode) of the MeOH extract of “Marrone di Roccadaspide” PGI chestnuts.

The accurate analysis of LC-MS profiles of burs, leaves, and chestnuts highlighted the occurrence of polar lipids that, on the basis of their accurate masses and characteristic fragmentation patterns, could be assigned to different chemical classes (e.g., phospholipids, glycolipids, sphingolipids), each one characterized by a specific polar moiety (“head group”) and differing inside by the various composing molecular moieties, which are structurally defined by fatty acids or other hydrocarbon portions varying in chain length and in saturation degree. To the best of our knowledge, this is the first report of polar lipids in chestnuts and by-products of *C. sativa* (Marrone di Roccadaspide). Noteworthy, the MeOH extract of chestnuts showed the widest range of polar lipids, so in order to allow their more effective separation and identification, a different chromatographic method was used for its analysis (Figure 4; Table 4).

Among phospholipids, metabolites ascribable to lyso-phosphatidylinositols (l-PI) and phosphatidylinositols (PI), lyso-phosphatidic acids (l-PA), lyso-phosphatidylglycerols (l-PG), lyso-phosphatidylcholines (l-PC), and N-acylglycerophosphatidylethanolamines (NA-GPE), could be putatively identified both in burs and leaves and in chestnuts (Table 3 and Table 4), while phosphatidylethanolamines (PE) and lyso-phosphatidylethanols (l-PEth) could be detected only in chestnuts (Table 4). In particular, the analysis of chestnuts LC-ESI/MSMS spectra revealed the presence of both l-PI and PI species, being characterized by the diagnostic product ion at *m*/*z* 241 Da, corresponding to the dehydrated form of the inositol-phosphate, and by the [(M-162)-H]^−^ and [(M-180)-H]^−^ product ions formed by neutral loss of the inositol moiety (Table 4) [21]. Moreover, the occurrence of lipids ascribable to l-PA (**103**, **105**, **107**, **111**) and l-PG (**101**, **102**, **130**) classes could be ascertained by the occurrence of diagnostic product ions at *m*/*z* 153 and 245 Da corresponding to the mono-dehydrated form of the glycerol phosphate and glycerolphosphoglycerol anions, respectively, and by the occurrence, in l-PGs spectra, of product ions formed by neutral losses of 92 and 74 Da, corresponding to glycerol and its mono-dehydrated form, respectively (Table 4). In agreement with literature data, describing the formation, in negative ion mode, of [(M+FA)−H]^−^ formic acid adducts for phosphatidylcholine derivatives, the observation in tandem mass spectrum of compound **104** of a product ion formed by the neutral loss of 60 Da from [(M+FA)−H]^−^ allowed identifying this molecule as l-PC. This product ion could be attributed to the [(M-15)−H]^−^ ion, in which the l-PC derivative lost a methyl group from the choline head group to generate formic acid methyl ester [21]. Compounds **144**, **146**, **147**, **150**, and **151** showed the typical fragmentation pattern of PE, in particular being characterized by the occurrence of product ions formed by the neutral loss of one fatty acid, whole or monodehydrated, and by fatty acid anions, whose ion intensity ratio allowed establishing the acyl residue regiospecificity (Table 4) [21]. The analysis of accurate masses and fragmentation patterns of compounds **95** and **129**, ascertaining the occurrence of a main product ion (*m*/*z* 402 and 404, respectively) corresponding to the phosphorylated N-fatty amide head group ion ([RCONHCH_2_CH_2_OPO_3_H]^−^ ion), allowed defining them as NA-GPEs, in which the fatty acid is N-acylated on the nitrogen of the ethanolamine head group (Table 4) [21]. Finally, the analysis of the tandem mass spectra of compounds **136**, **137**, and **140** allowed identifying in chestnuts MeOH extract the occurrence of metabolites ascribable to the class of lyso-glycerophosphatidylethanol (l-PEth), which were characterized by the diagnostic product ion at *m*/*z* 181 corresponding to the mono-dehydrated form of glycero-phosphatidylethanol (Table 4) [28,29].

Furthermore, the careful study of mass spectrometric data suggested the occurrence of different classes of glycolipids in chestnuts and in burs and leaves, with the first displaying the highest number (Table 3 and Table 4). So, compounds **92**, **97**, **98**, and **131** could be identified as sulfoquinovosyl-monoacylglycerols (SQMG) and compounds **118**, **121**, **142**, **143**, and **145** could be identified as sulfoquinovosyldiacylglycerols (SQDG) (Table 3 and Table 4), which are glycolipids composed of a glycerol backbone acylated at *sn*-1 and/or *sn*-2 positions with fatty acyl moieties showing various degrees of unsaturation, and of a polar head group represented by a sulfoquinovose molecule originating, in MSMS spectra, the class-diagnostic product ion at *m*/*z* 225, for which a structure bearing an epoxydic bridge between carbon 1 and 2 of the quinovosylic ring has been recently proposed [21]. Moreover, the analysis of LC-MS/MS data allowed assigning compounds **100**, **133–135** as digalactosylmonoacylglycerols (DGMG), and compounds **123**, **125**, **126**, and **148** as digalactosyldiacylglycerols (DGDG) [21,30,31], by considering, among others, the presence in their spectra of the characteristic product ions at *m*/*z* 415 and 397, corresponding to the digalactosylglycerol anion in the whole and in monodehydrated form, respectively (Table 3 and Table 4). Analogously, compounds **138**, **139**, and **141**, occurring only in chestnut extract, could be identified as monogalactosylmonoacylglycerols (MGMG) on the basis of the occurrence in their tandem mass spectra of the product ions at *m*/*z* 253 and 235 corresponding to the glycerol anion, in whole or monodehydrated form, which was glycosylated with only one sugar unit (Table 4) [21,30].

Finally, the analysis of full and tandem mass spectra highlighted the occurrence of an additional polar lipid class, i.e., the sphingolipids, which were chemically composed of a sugar moiety, usually glucose, glycosylated to an amino alcohol long chain base, inturn, *N*-acylated with a fatty acid generally made up of 14–26 carbon atoms and usually hydroxylated at C-2 [14]. In agreement with the literature data, the fragmentation pattern of compounds **122**, **123**, **125**, and **129**, which were characterized by the occurrence of abundant [(M-180)−H]^−^ and [(M-162)−H]^−^ product ions, originated by the neutral loss of a whole or mono-dehydrated hexose unit, along with minor product ions corresponding, e.g., to a fatty acyl chain linked to part of the long chain base, allowed defining them as glycosylceramides (GlyCer) (Figure 4, Table 3 and Table 4) [21,32].

### 3.8. Quantitative Analysis by LC-ESI/QTrap/MS/MS

For the most representative compounds, a LC-ESI/QTrap/MS/MS analysis, using MRM mode, has been carried out. In detail, MRM (Multiple Reaction Monitoring) is a tandem mass spectrometric technique in which a specific transition from a precursor ion to a product ion is monitored for each compound, assuring a very high selectivity and sensitivity (Table S1). On the basis of the fragmentation pattern, the specific MRM transition of each tested compound was selected (Table 5). In particular, for the [M-H]^−^ pseudomolecular ion at *m*/*z* 317 (crenatin, **7**), the transition 317 → 155 originated by the neutral loss of a glucose moiety (162 Da) was chosen for MRM analysis. For chestanin (**31**), which showed an M-H]^−^ pseudomolecular ion at *m*/*z* 937, the transition at *m*/*z* 467, due to the simultaneous loss of the glucose unit, the 3,4,5-trihydroxybenzyl alcohol unit, and the galloyl unit has been selected. The mass tandem spectrum of the [M-H]^−^ pseudomolecular ion at *m*/*z* 469 (cretanin, **32**) showed a very intense [(M-300)-H]^−^ product ion at *m*/*z* 169, which was ascribable to the galloyl moiety and originated by the neutral loss of both the glucose moiety and the 3,4,5-trihydroxybenzyl alcohol unit. The flavonoids quercetin-3-*O*-β-D-glucopyranoside (**54**) and quercetin-3-*O*-α-L-rhamnopyranoside (**63**), respectively corresponding to the [M-H]^−^ pseudomolecular ions at *m*/*z* 463 and 447, yielded characteristic fragmentation patterns in which the product ion was produced by the neutral loss of the sugar moiety, the glucose unit (neutral loss of 162 Da), and the rhamnose unit (neutral loss of 146 Da), respectively. For isorhamnetin-3-*O*-β-D-glucopyranoside (**61**), the transition 477 → 301 has been selected, showing the simultaneous loss of sugar moiety and methylene. The transition 301 → 284 due to the neutral loss of the radical OH• from the [M-H]^−^ pseudomolecular ion at *m*/*z* 301 was chosen for ellagic acid (**60**). Finally, for bartogenic acid (**96**), the main base peak at *m*/*z* 455 originated by the neutral loss of both a carbon dioxide molecule (44 Da) and a water molecule (18 Da) (Table 5) was selected.

## 4. Discussion

### 4.1. Evaluation of Antioxidant and Anti-Inflammatory Activity

With the aim to obtain preliminary information about the phenolic, tannin, and flavonoid content of MeOH extracts of burs, leaves, and chestnuts, analyses by spectrophotometric assays were carried out, highlighting very high contents for burs, high contents for leaves, and not detectable contents for chestnuts (Table 1).

The evaluation of the antioxidant activity by TEAC, DPPH, and FRAP assays confirmed this trend, evidencing the highest antioxidant activity for the MeOH extract of burs, followed by leaves extract, with a very low activity for chestnut extract (Table 2).

In order to confirm the antioxidant activity observed in the spectrophotometric assays, an in vitro antioxidant assay, using a non-toxic concentration of 5 µg/mL, was carried out. The leaves and burs extracts reduced intracellular levels of ROS, which were produced after stimulation of the THP-1-XBlue-MD2-CD14 cells with PCN, in a percentage of 58.84% ± 2.86% and 74.30% ± 2.96%, respectively, showing an antioxidant activitysimilar or higher than quercetin (66.41% ± 2.58%), which was used as a reference compound (Figure 1). Thus, a similar trend to that observed in the spectrophotometric assays was observed, with the burs extract showing the highest antioxidant activity. These results are in agreement with previous studies on *C. sativa* by-products [7,8,11].

Antiradical properties of analyzed extracts may decrease the LPS-induced intracellular inflammatory pathway just before lipid-derived aldehydes are created.

Antiradical agents could decrease the NF-κB-dependent production of pro-inflammatory markers and prevent LPS toxicity. With the aim to evaluate the anti-inflammatory activity of tested extracts, an in vitro assay for intracellular nuclear factor NF-κB activity determination was performed. NF-κB exists in the cytoplasm in an inactive complex of p50/p65 heterodimer bound to the inhibitory protein IκBs. After NF-κB activation, the enzyme IκB kinase phosphorylates the IκBα protein, causing the dissociation of IκBα from NF-κB. After activation, NF-κB is translocated into the nucleus, where it binds DNA and activates gene transcription [11].

Numerous stimuli, such as lipopolysaccharide (LPS), activate NF-κB and consequently cause the intracellular generation of ROS, which is one possible reason for cytotoxicity and the development of inflammation. MeOH extracts of leaves and burs at 5 µg/mL reduced NF-κB activation to 35.31% and 62.73%, respectively, showing an activity higher than prednisone (63.01% at 1 µM), which was used as a reference compound (Figure 2). Moreover, the level of the main pro-inflammatory second messenger NO was evaluated by in vitro assay showing a similar trend (Figure 2). The leaves extract resulted the most active extract in reducing NF-κB activation and NO production.

### 4.2. LC-MS Analysis of Specialized Metabolites and Polar Lipids in C. sativa Burs, Leaves, and Chestnuts

The careful analysis of LC-ESI/LTQOrbitrap/MS/MS of MeOH extracts of burs, leaves, and chestnuts allowed assigning putatively a wide range of compounds belonging to phenolic compounds such as ellagitannins, galloyl glucose derivatives, ellagic acid and phenol glucoside derivatives, flavonoids glycosides, as well as triterpenoids and polar lipids compounds.

In particular, mass spectrometric data analysis highlighted a similar composition of specialized metabolites in burs and leaves, with both extracts showing a high number, higher in the former, of hydrolyzable tannins and flavonoids, despite chestnuts for which only ellagic acid was identifiable as a phenolic compound. Considering that for these compounds, various biological activities such as antioxidant, antiviral, and antitumor activities have been reported [33,34], our results suggest a possible re-use of these by-products as a natural source of bioactive phytochemicals. Moreover, although some compounds were already reported in the burs and leaves of *C. sativa* [8,11,12], to the best of our knowledge, this is the first report on the detailed phenolic composition of *C. sativa* leaves.

Furthermore, a wide range of compounds belonging to different polar lipid classes, such as phospholipids, glycolipids, and sphingolipids, could be identified both in burs and leaves and in chestnuts, with the latter showing the higher number and variety, e.g., displaying metabolites belonging to PE, l-PEth, and MGMG classes that are not detectable in the other extracts; thereby, for the first time, the detailed and comprehensive characterization of the polar lipids in *C. sativa* chestnuts, burs, and leaves has been afforded. On the basis of the biological activities reported for lipid classes and their effects on human health, these data support the use of chestnuts in human nutrition as a food rich in different classes of bioactive and healthy lipids with beneficial effects [31,35,36,37].

### 4.3. Quantitative Analysis of Phenolic Compounds in Burs and Leaves

Quantitative analysis allowed us to determine the amount (mg/100 g dry weight) of representative compounds in the MeOH extract of *C. sativa* burs and leaves (Table 5). Cretanin (**32**) was the most abundant compound in both extracts with a concentration of 95.20 mg/100 g and 16.75 mg/100 g in leaves and burs, respectively. Moreover, the quantitative analysis of MeOH extract of leaves showed also a good content of chestanin (**31**) (85.84 mg/100 g) and isorhamnetin 3-*O*-β-*D*-glucopyranoside (**61**) (50.33 mg/100 g). About the other quantified compounds, burs and leaves showed similar amounts (mg/100 g) in dried MeOH extracts (Table 5).

## 5. Conclusions

The Italian “Marrone di Roccadaspide” is a labeled PGI product of the Campania region, representing an important economic resource. With the aim of exploiting its by-products for the development of nutraceutical and/or cosmetic formulations, the antioxidant and anti-inflammatory in vitro activities of MeOH extracts of burs and leaves of *C. sativa*, cultivar “Marrone di Roccadaspide”, have been evaluated and compared with those of chestnut extract, resulting higher than this latter.

In order to correlate the antioxidant and anti-inflammatory activity exhibited by both by-product extracts to their chemical composition, and to investigate the chemical composition of the less active chestnut extract, a comprehensive chemical analysis based on LC-ESI/LTQOrbitrap/MS/MS has been carried out on all tested extracts. In this way, and in agreement with the results of Folin–Ciocalteu assay highlighting a high phenolic content only in burs and leaves, a large number of compounds belonging to the five main classes of specialized bioactive metabolites such as hydrolyzable tannins, glycosylated flavonoids, ellagic acid and phenol glucoside derivatives, and triterpenoids were identified in by-product extracts but not in chestnut extracts. It is noteworthy that this latter was shown to be characterized by a wide range of polar lipid classes, such as phospholipids, glycolipids, and sphingolipids, which were putatively identified for the first time through a detailed and comprehensive analysis.

Hydrolyzable tannins represent a class of molecules with a wide range of biological activities [38]. The free radical scavenging activity, tested by TEAC and DPPH assays, is higher in the burs extract, and this evidence could be correlated to its higher tannins and flavonoids contents. Leaves extract if compared to burs extract is characterized by lower tannins and flavonoids content, but by a higher amount of polar lipids. Considering the higher ferric reducing antioxidant power of the burs extract, once again, it is evident how the tannins and flavonoids content is responsible for the antioxidant activity. The results obtained by the quantitative analysis of burs and leaves extracts highlights a higher content of some specialized metabolites (Table 5) in the MeOH extracts of the leaves and, thus, reinforce the notion that the content of tannin (higher in the burs) accounts for its higher antioxidant activity.

On the other hand, the highest anti-inflammatory activity, measured as the ability to reduce both NF-κB activation and NO production, has been observed for the leaves extract. These data suggest how other metabolites along with tannins can contribute to anti-inflammatory activity. MeOH extracts of leaves showed a chemical profile rich in tannins, other phenolic compounds as well as polar lipids. Polar lipids have been related to anti-inflammatory activities, and glycolipids containing unsaturated MGDG have been associated to anti-inflammatory effects exerted by the inhibition of NO production [39]. Moreover, also, lysophospholipids are recognized as important cellular signaling molecules and are involved in important processes including inflammation mediated by l-PL-specific G-protein coupled receptor [21]. According to these activities, the mild anti-inflammatory activity shown by chestnuts extract could be justified by polar lipids, while the synergism between phenolic compounds and polar lipids, occurring in the leaves, could be responsible for its higher anti-inflammatory activity.

Altogether, these results represent an important step to both encourage the recycling and exploitation of *Castanea* by-products, favoring the circular economy and reducing the environmental impact related to the management of chestnut grove waste, and to support the use of chestnuts in human nutrition [31,35,36,37].

## Figures and Tables

**Table 1:** Total phenol, total tannin and total flavonoid content of MeOH extracts of *C. sativa* (Marrone di Roccadaspide) burs, leaves, and chestnuts.

*C. sativa* MeOH Extracts	Total Phenol Content (mg GAE/g ± SD ^a^)	Total Tannin Content (mg GAE/g ± SD ^a^)	Total Flavonoid Content (mg rutin/g ± SD ^a^)
burs	580.44 ± 29.63	276.44 ± 13.52	87.19 ± 1.98
leaves	298.96 ± 14.81	105.25 ± 9.95	45.54 ± 0.99
chestnuts	N.D.	N.D.	N.D.

^a^ SD: Standard deviation of three independent experiments.

**Table 2:** Antioxidant activity of MeOH extracts of *C. sativa* (Marrone di Roccadaspide) burs, leaves, and chestnuts evaluated by Trolox Equivalent Antioxidant Capacity (TEAC), 1,1-diphenyl-2-picrylhydrazyl (DPPH), and Ferric Reducing Antioxidant Power (FRAP) assays.

*C. sativa* MeOH Extracts	Free Radical Scavenging Activity (DPPH) EC_50_ (µg/mL ± SD ^a^) ^b^	(TEAC) (mg/mL ± SD ^a^) ^c^	FRAP (mmol ferric sulfate/g ± SD ^a^) ^d^
burs	4.21 ± 0.01	3.03 ± 0.03	2.96 ± 0.02
leaves	3.06 ± 0.01	3.01 ± 0.02	1.48 ± 0.02
chestnuts	34.64 ± 0.01	0.57 ± 0.01	0.18 ± 0.02

^a^ SD: Standard deviation of three independent experiments. ^b^ Determined by DPPH test. ^c^ Determined by TEAC assay ^d^ Determined by FRAP assay.

**Table 3:** Metabolites putatively identified in the MeOH extract of *C. sativa* (Marrone di Roccadaspide) burs (CSB) and leaves (CSL).

N.	Compound	*R_t_* (min) ^a^	Molecular Formula	[M-H]^− a^	[M-2H]^2− a^	[(M+FA)-H]^− a^	Delta (ppm) ^a^	Product ions (*m*/*z*) ^a^	CSB	CSL
**1**	Sucrose	5.10	C_12_H_22_O_11_	341.1083		387.1137	1.38	179, 161	X	X
**2**	Sorbitol/glucitol	5.22	C_6_H_14_O_6_			227.0766	2.05	181	X	-
**3**	Monogalloyl glucose	6.44	C_13_H_16_O_10_	331.0665			1.59	313, 271, 211, 193, 169, 125	X	X
**4**	NHTP-glucose (vescalin)	7.20	C_27_H_20_O_18_	631.0561			−0.69	613, 587, 569, 551, 467, 441, 425	X	X
**5**	HHDP-glucose isomer	7.27	C_20_H_18_O_14_	481.0610			−0.61	421, 301, 275	X	X
**6**	HHDP-glucose isomer	7.60	C_20_H_18_O_14_	481.0601			−0.48	421, 301, 275	X	-
**7**	Phenol glucoside (crenatin) ^#^	7.88	C_13_H_18_O_9_	317.0872			1.61	299, 155	X	X
**8**	Digalloyl glucose isomer	8.31	C_20_H_20_O_14_	483.0771			−0.87	465, 331, 313, 169	X	X
**9**	Hexahydroxydiphenic acid	8.55	C_14_H_10_O_10_	337.0192			0.44	293, 249, 169, 167	X	-
**10**	Digalloyl glucose isomer	11.07	C_20_H_20_O_14_	483.0772			0.58	465, 331, 313, 169	X	-
**11**	Digalloyl glucose isomer	12.29	C_20_H_20_O_14_	483.0763			−1.33	465, 331, 313, 169	X	-
**12**	NHTP-valoneoyl-glucose (castavaloninic acid)	14.01	C_48_H_30_O_31_	1101.0671	550.0282		−1.44	1057, 933, 931, 631, 587, 449, 441, 425	X	-
**13**	Castacrenin C	14.05	C_27_H_18_O_17_	613.0456			−0.71	595, 493, 301, 299	X	-
**14**	Castacrenin B	14.87	C_27_H_18_O_17_	613.0456			−0.71	595, 493, 301, 299	X	-
**15**	Chebulic acid isomer	14.94	C_14_H_12_O_11_	355.0297			0.45	337, 311, 267, 191	X	-
**16**	Chebulic acid isomer	15.45	C_14_H_12_O_11_	355.0296			0.04	337, 311, 267, 191	X	-
**17**	NHTP-HHDP-glucose (vescalagin)	15.67	C_41_H_26_O_26_	933.0611	466.0271		−1.88	915, 631, 613, 587, 569, 551, 467, 441, 425, 301	X	-
**18**	Castamollissin/ maplexin D	15.73 ^b^	C_20_H_20_O_13_		467.0821 ^b^		0.28 ^b^	449, 423, 315, 169	-	X
**19**	Castacrenin A	15.97	C_27_H_18_O_17_	613.0453			−1.10	595, 523, 493, 301, 299	X	-
**20**	Bis-HHDP-glucose isomer (casuariin/pedunculagin isomer)	16.45	C_34_H_24_O_22_	783.0669	391.0299		−0.88	765, 481, 301, 275	X	X
**21**	Chesnatin	16.68	C_27_H_26_O_18_	637.1030			−0.82	593, 467, 305	X	-
**22**	HHDP-Valoneoyl-glucose isomer (praecoxin A/platycariin isomer)	17.13	C_41_H_28_O_27_	951.0714	475.0315		−2.11	907, 783, 465, 453	X	-
**23**	Galloyl-HHDP-glucose isomer (punicacortein A-B/pterocaryanin B/gemin D)	17.46	C_27_H_22_O_18_	633.0718	316.0328		−0.76	615, 589, 481, 463, 301, 275, 257	X	-
**24**	Bis-HHDP-glucose isomer (pedunculagin/casuariin isomer)	17.56	C_34_H_24_O_22_	783.0672	391.0298		−0.42	765, 721, 481, 301, 275	X	-
**25**	Trigalloyl glucose isomer	17.71	C_27_H_24_O_18_	635.0878			−0.08	483, 465, 331, 313, 271, 211	X	-
**26**	Galloyl-chebuloyl-HHDP-glucose (chebulagic acid)	18.03	C_41_H_30_O_27_	953.0881	476.0402		−1.03	909, 785	X	-
**27**	Isochesnatin	18.05	C_27_H_26_O_18_	637.1030			−0.80	593, 469	X	-
**28**	Digalloyl-HHDP-glucose isomer (1-desgalloyleugeniin isomer)	18.64	C_34_H_26_O_22_	785.0834	392.0372		0.22	633, 615, 483, 313, 301, 275	X	X
**29**	Galloyl-bis-HHDP-glucose (stachyurin)	18.71	C_41_H_28_O_26_	935.0787	467.0347		0.25	917, 783, 633	X	-
**30**	Methylvaloneoyl–NHTP–glucose (vescavaloninic/castavaloninic acid methyl ester)	18.86	C_49_H_32_O_31_	1115.0844			−0.01	1053, 933, 569	X	-
**31**	Galloyl phenol glucoside dimer (chestanin) ^#^	19.21	C_40_H_42_O_26_	937.1825	468.0897		−0.59	893, 637, 635, 467, 301	X	X
**32**	Galloyl phenol glucoside (cretanin) ^#^	19.22	C_20_H_22_O_13_	469.0974			−0.55	169	X	X
**33**	Galloyl-chebuloyl-HHDP-glucose (chebulagic acid)	19.25	C_41_H_30_O_27_	953.0881			0.98	909, 785	X	-
**34**	Trigalloyl glucose isomer	19.41	C_27_H_24_O_18_	635.0870			−1.34	483, 465, 331, 313, 271, 211	X	-
**35**	Digalloyl-HHDP-glucose isomer (tellimagrandin I isomer)	19.97	C_34_H_26_O_22_	785.0825	392.0377		−0.69	633, 615, 483, 313, 301, 275	X	X
**36**	Trigalloyl glucose isomer	20.20	C_20_H_22_O_13_	635.0873			0.75	483, 465, 313, 271, 211	X	X
**37**	3-O-SSupp-coumaroylquinic acid	20.21	C_16_H_18_O_8_	337.0920			0.55	191, 179	X	-
**38**	Galloyl phenol glucoside	20.58 ^b^	C_20_H_22_O_13_	469.0980 ^b^			0.75 ^b^	169	-	X
**39**	coumaroylquinic acid	20.72	C_16_H_18_O_8_	337.0919			0.37	191, 179	X	X
**40**	Galloyl-methylchebuloyl-HHDP-glucose (chebulagic acid methyl ester)	20.74	C_42_H_32_O_27_	967.1033	483.0481		−1.50	785, 765	X	X
**41**	Galloyl phenol glucoside dimer (isochestanin)	20.75 ^b^	C_40_H_42_O_26_	937.1877 ^b^			−0.40 ^b^	637, 467	-	X
**42**	Ellagic acid pentoside	20.84	C_19_H_14_O_12_	433.0402			0.09	301, 300	X	X
**43**	Euphorbin A/B	21.15	C_82_H_58_O_53_		944.0816			1419, 922, 860, 783, 467, 301	X	-
**44**	Nobotanin A/Malabathrin B/1-Desgalloylrugosin F/Zeylaniin A isomer	21.35	C_75_H_52_O_48_		859.0722		0.41	1417, 1115, 1085, 937, 917, 783, 767, 301	X	-
**45**	Galloyl-methylchebuloyl-HHDP-glucose (chebulagic acid methyl ester)	21.44	C_42_H_32_O_27_	967.1038	483.0478		−0.99	785, 765	X	X
**46**	Rugosin E/Camptothin B	21.56	C_75_H_54_O_48_		860.0796		−1.40	1419, 1089, 953, 937, 935, 917, 909, 785, 767, 699, 615, 597, 465, 301, 275, 249	X	-
**47**	Digalloyl-HHDP-glucose isomer (tellimagrandin I isomer)	21.77	C_34_H_26_O_22_	785.0833	392.0378		0.03	633, 615, 483, 313, 301	X	X
**48**	Quercetin galloyl hexoside	21.99	C_28_H_24_O_16_	615.0969			−1.95	463, 301	X	X
**49**	Valoneic acid dilactone	22.21	C_21_H_10_O_13_	469.0035			−0.55	425, 301, 299, 169	X	-
**50**	Rugosin E/Camptothin B	22.42	C_75_H_54_O_48_		860.0798		1.26	1419, 1089, 953, 937, 935, 917, 909, 785, 767, 699, 615, 597, 465, 301, 275, 249	X	-
**51**	Isorhamnetin deoxyhexose hexoside	22.52	C_28_H_32_O_16_	623.1603			−0.61	315, 300, 271	X	X
**52**	Kaempferol deoxyhexose hexoside	22.57	C_27_H_30_O_15_	593.1500			−0.13	285	X	X
**53**	Methylellagic acid hexoside	22.62 ^b^	C_21_H_18_O_13_	477.0662 ^b^			−0.24 ^b^	315, 301	-	X
**54**	Quercetin 3-*O*-β-D-glucopyranoside ^#^	22.74	C_21_H_20_O_12_	463.0869			−0.19	301	X	X
**55**	Quercetin hexuronoside	22.80	C_21_H_18_O_13_	477.0660			−0.75	301	X	-
**56**	Galloyl phenol glucoside gallate (galloyl-cretanin)	22.97	C_27_H_26_O_17_	621.1082			−0.38	577, 469, 451, 317, 313	X	X
**57**	Trigalloyl-HHDP-glucose (Tellimagrandin II)	23.05	C_41_H_30_O_26_	937.0933	468.0430		0.03	MS^2^ (468): 767, 635, 633, 617, 392 [M−2H−152]^2−^, 313, 301, 169	X	X
**58**	Tetragalloyl glucose isomer	23.14	C_34_H_28_O_22_	787.0987	393.0453		−0.14	635, 617, 483, 465, 447, 295	X	X
**59**	Kaempferol hexoside (astragalin)	23.97	C_21_H_20_O_11_	447.0922			0.07	327, 285	X	X
**60**	Ellagic acid ^#^	24.37	C_14_H_6_O_8_	300.9989			0.47	284, 257, 229, 201, 185, 145	X	X
**61**	Isorhamnetin 3-*O*-β-D-glucopyranoside ^#^	24.60	C_22_H_22_O_12_	477.1029			0.33	357, 315, 314	X	X
**62**	Quercetin pentoside	24.68 ^b^	C_20_H_18_ O_11_	433.0769 ^b^			0.77 ^b^	301, 300	-	X
**63**	Quercetin 3-*O*-α-L-rhamnopyranoside ^#^	25.06 ^b^	C_21_H_20_O_11_	447.0924 ^b^			0.50 ^b^	301	-	X
**64**	Methyl coumaroyl quinate	25.11	C_17_H_20_O_8_	351.1082			2.29	163	X	X
**65**	Methylellagic acid pentoside	25.51	C_20_H_16_O_12_	447.0553			−1.12	315, 301, 300	X	X
**66**	Isorhamnetin hexuronoside	26.05	C_22_H_20_O_13_	491.0818			−0.40	315, 301	X	X
**67**	Lignan hexoside	27.01	C_26_H_32_O_11_	519.1862			0.14	357	X	X
**68**	Valoneic acid dilactone methyl ester	27.14	C_22_H_12_O_13_	483.01902			−0.53	451, 301	X	X
**69**	Bartogenic acid hexoside	27.33	C_36_H_56_O_12_	679.3693		725.3736	0.67	559, 517, 455	X	-
**70**	Lignan hexoside	27.34	C_26_H_34_O_11_	521.2018		567.2074	0.19	359, 341, 177	X	X
**71**	Dimethylellagic acid pentoside	27.53	C_21_H_18_O_12_	461.0713			−0.35	446, 328, 313, 299, 285, 284	X	X
**72**	Quercetin coumaroyl hexoside	29.43	C_30_H_26_O_14_	609.1234			−0.77	463, 301	X	X
**73**	Dimethylellagic acid deoxyhexoside	29.70	C_22_H_20_O_12_	475.0865			−1.23	460, 328, 313, 299, 275, 217, 193	X	X
**74**	Trimethylellagic acid hexoside	30.74	C_23_H_22_O_13_	505.0981		551.1031		343, 328, 313, 299, 284	X	X
**75**	Kaempferol coumaroyl hexoside	32.45	C_30_H_26_O_13_	593.1285			−0.72	447, 285, 257, 229	X	*-*
**76**	Kaempferol coumaroyl hexoside	32.87	C_30_H_26_O_13_	593.1285			−0.72	447, 285, 257, 229	X	*-*
**77**	Kaempferol deoxyhexosyl coumaroyl hexoside	33.27 ^b^	C_36_H_36_O_17_	739.1870 ^b^			0.18 ^b^	593, 575, 453, 285	-	X
**78**	Isorhamnetin coumaroyl hexoside	33.29	C_31_H_28_O_14_	623.1394			−0.21	477, 315, 300	X	X
**79**	Kaempferol coumaroyl hexoside	34.02	C_30_H_26_O_13_	593.1284			−0.92	447, 285, 257, 229	X	X
**80**	Castaartancrenoic acid D/E hexoside	34.17 ^b^	C_36_H_58_O_10_	649.3951 ^b^		695.3998 ^b^	0.67 ^b^	649, 487	-	X
**81**	Kaempferol coumaroyl hexoside	34.57	C_30_H_26_O_13_	593.1285			−0.82	447, 285, 257, 229	X	X
**82**	Roburgenic acid isomer	35.58	C_30_H_46_O_8_	533.3109			0.03	485, 471, 457, 453	X	-
**83**	Isorhamnetin coumaroyl hexoside	37.04 ^b^	C_31_H_28_O_14_	623.1395 ^b^			−0.10 ^b^	477, 315, 300	-	X
**84**	Dimethylellagic acid	38.17	C_16_H_10_O_8_	329.0296			1.33	314, 299, 285	X	-
**85**	Trimethylellagic acid deoxyhexoside	38.51	C_23_H_22_O_12_			535.1085	0.43	343, 328, 313, 299	X	-
**86**	l-PI (18:3)	39.28	C_27_H_47_O_12_P	593.2720			−0.18	413, 315, 277, 241	X	X
**87**	Kaempferol acetyl coumaroyl hexoside	39.33	C_32_H_28_O_14_	635.1392			−0.58	575, 489, 285	X	X
**88**	Castaartancrenoic acid B	39.33	C_27_H_44_O_5_	447.31039			−0.25	429, 401, 365	X	-
**89**	Dimethylellagic acid	39.60	C_16_H_10_O_8_	329.0297			1.42	314, 299, 285	X	-
**90**	l-PI (18:2)	40.20	C_27_H_49_O_12_P	595.2876			−0.84	415, 315, 279, 241, 179	X	X
**91**	Quercetin dicoumaroyl hexoside	40.21	C_39_H_32_O_16_	755.1605			−0.19	609, 463, 301	X	X
**92**	SQMG (18:3)	40.32	C_27_H_46_O_11_S	577.2676			−0.22	299, 277, 225	X	X
**93**	l-PI (16:0)	40.43	C_25_H_49_O_12_P	571.2873			−0.77	409, 391, 333, 315, 255, 241, 223, 171	X	X
**94**	Roburgenic acid isomer	40.43	C_30_H_46_O_8_	533.3104			−0.89	485, 471	X	-
**95**	NA-GPE (18:2)	40.45	C_23_H_44_O_7_NP	476.2767			−0.98	415, 279, 214, 196, 153	X	X
**96**	Bartogenic acid ^#^	40.78	C_30_H_46_O_7_	517.3156			−0.72	499, 455, 437	X	-
**97**	SQMG (18:2)	41.37	C_27_H_48_O_11_S	579.2831			−0.48	299, 279, 225	X	X
**98**	SQMG (16:0)	41.55	C_25_H_48_O_11_S	555.2833			−0.05	299, 255, 225	X	X
**99**	Kaempferol dicoumaroyl hexoside	41.81	C_39_H_32_O_15_	739.1650			−1.04	593, 575, 453, 285,	X	X
**100**	DGMG (18:3)	42.16	C_33_H_56_O_14_	675.3594			1.19	415, 397	X	X
**101**	l-PG (16:0)	42.29	C_22_H_45_O_9_P	483.2722			0.92	391, 255, 245, 227, 153	X	X
**102**	l-PG (18:1)	43.32 ^b^	C_24_H_47_O_9_P	509.2876 ^b^			0.34 ^b^	417, 281, 245, 227, 153	-	X
**103**	l-PA (18:3)	43.78	C_21_H_37_O_7_P	431.2194			0.24	413, 277, 153	X	X
**104**	l-PC (16:0)	44.46	C_25_H_52_O_9_NP			540.3298	0.18	480, 255, 225	X	X
**105**	l-PA (18:2)	44.94	C_21_H_39_O_7_P	433.2350			−1.15	171, 153	X	X
**106**	Kaempferol acetyl dicoumaroyl hexoside	45.04	C_41_H_34_O_16_	781.1753			−0.98	635, 617, 495, 435, 285	X	X
**107**	l-PA (16:0)	45.26	C_19_H_39_O_7_P	409.2354			1.01	391, 255, 153	X	X
**108**	Kaempferol acetyl dicoumaroyl hexoside	45.46	C_41_H_34_O_16_	781.1757			−0.76	635, 617, 495, 435, 285	X	X
**109**	Kaempferol acetyl dicoumaroyl hexoside	45.87	C_41_H_34_O_16_	781.1754			−0.92	635, 617, 495, 435, 285	X	X
**110**	Kaempferol acetyl dicoumaroyl hexoside	46.21	C_41_H_34_O_16_	781.1767			0.18	635, 617, 495, 435, 285	X	-
**111**	l-PA (18:1)	46.36	C_21_H_41_O_7_P	435.2510			0.83	417, 281, 153	X	X
**112**	Trimethylellagic acid	46.43	C_17_H_12_O_8_	343.0452			0.31	328, 313, 299, 297, 284, 275	X	-
**113**	Kaempferol diacetyl dicoumaroyl hexoside	47.87	C_43_H_36_O_17_	823.1856			−1.31	677, 659, 635, 617, 557, 531, 391, 285	X	X
**114**	Kaempferol diacetyl dicoumaroyl hexoside	48.04	C_43_H_36_O_17_	823.1865			−0.50	677, 659, 617, 391, 285	X	X
**115**	Kaempferol diacetyl dicoumaroyl hexoside	48.34	C_43_H_36_O_17_	823.1862			0.83	677, 659, 635, 617, 557, 531, 391, 285	X	X
**116**	Kaempferol diacetyl dicoumaroyl hexoside	48.57	C_43_H_36_O_17_	823.1864			−0.58	677, 659, 617, 557, 531, 391, 285	X	*-*
**117**	PI (16:0; 18:3)	51.26	C_43_H_77_O_13_P	831.5017			−0.11	575, 553, 413, 391, 277, 255	X	X
**118**	SQDG (16:0; 18:3)	51.58 ^b^	C_43_H_76_O_12_S	815.4966 ^b^			−0.90 ^b^	559, 537, 277, 255	-	X
**119**	Hederagenin	52.31	C_30_H_40_O_4_	471.3466			−0.50	453, 425, 407	X	X
**120**	2-Pentadecanone	52.72	C_16_H_32_O_3_			271.2270	0.92	225, 209	X	-
**121**	SQDG (16:0; 16:0)	53.21 ^b^	C_41_H_78_O_12_S	793.5132 ^b^			0.20 ^b^	537, 255, 225	-	X
**122**	GlyCer (t18:1;h16:0)	54.01	C_40_H_77_O_10_N	730.5461		776.5514	−0.40	568, 550, 326, 271	X	X
**123**	DGDG (18:3; 18:3)	54.94 ^b^	C_51_H_84_O_15_	935.5726 ^b^		981.5783 ^b^	−0.08 ^b^	657, 397, 341, 323, 277	-	X
**124**	GlyCer (d18:2;h16:0)	55.34	C_40_H_75_O_9_N	712.5351		758.54028	−0.99	550, 532, 312, 296, 271, 253, 225	X	X
**125**	DGDG (18:3, 16:0)	56.40	C_49_H_86_O_15_	913.5881		959.5929	2.03	657, 635, 379, 277	X	X
**126**	DGDG (18:2, 16:0)	57.29	C_49_H_88_O_15_	915.6023		961.6087	−1.81	659, 635, 379	X	X
**127**	Glycerol-ω-hydroxyacid-ferulic acid (22:0)	57.83	C_35_H_58_O_8_	605.4042			−0.92	531, 513, 193, 175	X	-
**128**	GlyCer (t18:1;h22:0)	58.78 ^b^	C_46_H_89_O_10_N	814.6398 ^b^			−0.62 ^b^	652, 634, 410, 355, 337, 309	-	X

^a^ Calculated from the LC-MS analysis of CSB in negative ion mode; ^b^ calculated from the LC-MS analysis of CSL in negative ion mode; ^#^ the identification of this compound was corroborated by comparison with standards.

**Table 4:** Metabolites putatively identified in the MeOH extract of *C. sativa* (Marrone di Roccadaspide) chestnuts.

N.	Compound	*R_t_* (min)	Molecular Formula	[M-H]^−^	[(M+FA)-H]^−^	Delta (ppm)	Product ions (*m*/*z*)
**60**	Ellagic acid ^#^	7.28	C_14_H_6_O_8_	300.9989		0.47	284, 257, 229, 201, 185, 145
**90**	l-PI (18:2)	19.06	C_27_H_49_O_12_P	595.2880		0.28	415, 315, 279, 241, 223
**93**	l-PI (16:0)	19.11	C_25_H_49_O_12_P	571.2883		0.95	391, 315, 255, 241, 223
**95**	NA-GPE (18:2)	19.28	C_23_H_44_O_7_NP	476.2774		0.43	402, 384, 214, 171, 153
**97**	SQMG (18:2)	19.62	C_27_H_48_O_11_S	579.2836		0.36	299, 279, 225
**98**	SQMG (16:0)	19.68	C_25_H_48_O_11_S	555.2839		1.05	299, 255, 225
**129**	NA-GPE (18:1)	19.89	C_23_H_46_O_7_NP	478.2931		0.57	404, 386, 171, 153
**130**	l-PG (18:2)	20.06	C_24_H_45_O_9_P	507.2719		0.26	415, 279, 245, 227, 153
**101**	l-PG (16:0)	20.17	C_22_H_45_O_9_P	483.2722		0.86	391, 255, 245, 227, 153
**131**	SQMG (18:1)	20.17	C_27_H_50_O_11_S	581.2994		0.64	563, 299, 281, 225
**100**	DGMG (18:3)	20.52	C_33_H_56_O_14_	675.3587	721.3639	0.10	397
**132**	Bartogenic acid ^#^	20.69	C_30_H_46_O_7_	517.3163		0.58	499, 455,
**102**	l-PG (18:1)	20.80	C_24_H_47_O_9_P	509.2878		0.81	417, 281,
**133**	DGMG (18:2)	21.20	C_33_H_58_O_14_	677.3751	723.3796	1.15	415, 397, 235
**134**	DGMG (16:0)	21.31	C_31_H_58_O_14_	653.3751	699.3802	1.19	415, 397,
**103**	l-PA (18:3)	21.65	C_21_H_37_O_7_P	431.2193		0.03	413, 153
**135**	DGMG (18:1)	22.11	C_33_H_60_O_14_	679.3905	725.3952	0.83	415, 397, 235
**104**	l-PC (16:0)	22.45	C_24_H_50_O_7_NP		540.3297	0.21	480, 255
**105**	l-PA (18:2)	22.51	C_21_H_39_O_7_P	433.2351		0.40	415, 279, 153
**107**	l-PA (16:0)	22.74	C_19_H_39_O_7_P	409.2352		0.50	391, 255, 153
**136**	l-PEth (18:2)	23.42	C_23_H_43_O_7_P	461.2664		0.33	415, 279, 181
**137**	l-PEth (16:0)	23.71	C_21_H_43_O_7_P	437.2661		−0.43	255, 181
**111**	l-PA (18:1)	23.77	C_21_H_41_O_7_P	435.2508		0.47	417, 391, 281, 153
**138**	MGMG (18:2)	24.97	C_27_H_48_O_9_	515.3218	561.3273	0.74	279, 253, 235
**139**	MGMG (16:0)	25.20	C_25_H_48_O_9_	491.3215	537.3274	0.08	255, 253, 235
**140**	l-PEth (18:1)	24.97	C_23_H_45_O_7_P	463.2822		0.55	281, 181
**141**	MGMG (18:1)	26.69	C_27_H_50_O_9_	517.3375	563.3439	0.68	281, 253, 235
**122**	GlyCer (t18:1; h16:0)	41.88	C_40_H_77_O_10_N	730.5461	776.5514	−0.40	568, 550, 326, 271
**142**	SQDG (16:0; 18:2)	43.48	C_43_H_78_O_12_S	817.5129		−0.10	723, 561, 537, 279, 225
**143**	SQDG (16:0; 18:2)	44.50	C_43_H_78_O_12_S	817.5147		2.06	723, 561, 537, 279, 255, 225
**144**	PE (18:3, 18:2)	44.88	C_41_H_72_O_8_NP	736.4924		1.70	458, 456, 279, 277, 255
**124**	GlyCer (d18:2; h16:0)	45.44	C_40_H_75_O_9_N	712.5363	758.5417	0.72	550, 532, 312, 296, 271, 253, 225
**145**	SQDG (16:0; 18:1)	46.33	C_43_H_80_O_12_S	819.5280		−0.80	563, 537, 281, 255, 225
**146**	PE (16:0; 18:3)	46.22	C_39_H_72_O_8_NP	712.4899		−1.76	456, 277, 255
**147**	PE (18:2; 18:2)	46.81	C_41_H_74_O_8_NP	738.5067		−0.16	476, 458, 279
**148**	DGDG (18:2; 18:2)	48.10	C_51_H_88_O_15_	939.6033	985.6087	−0.66	677, 659, 415, 397, 279
**149**	PG (16:0;18:2)	48.31	C_40_H_75_O_10_P	745.5022		1.06	507, 489, 483, 465, 415, 391, 279, 255
**150**	PE (16:0; 18:2)	48.85	C_39_H_74_O_8_NP	714.5063		−0.76	458, 434, 279, 255
**151**	PE (18:2; 18:1)	49.28	C_41_H_76_O_8_NP	740.5226		0.13	478, 476, 460, 458, 281, 279
**152**	MGDG (16:0; 12:2)	50.44	C_37_H_66_O_10_	669.4580		1.17	499, 431, 413, 255

^#^ The identification of this compound was corroborated by comparison with standards.

**Table 5:** Quantitative results of phenolic compounds occurring in MeOH extract of *C. sativa* (Marrone di Roccadaspide) burs and leaves.

Compound	Mrm Transition	R^2^	Regression Line	mg/100 g Burs ± SD *	mg/100 g Leaves ± SD *
Crenatin (**7**)	317→155	0.9967	y = 0.000143x + 0.0284	0.90 ± 0.09	15.77 ± 1.17
chestanin (**31**)	937→467	0.9962	y = 0.000228x + 0.000139	3.23 ± 0.06	85.84 ± 2.18
cretanin (**32**)	469→169	0.9969	y = 3.25e−5x-0.00466	16.75 ± 2.24	95.2 ± 7.21
quercetin 3-*O*-β-D-glucopyranoside (**54**)	463→301	0.9930	y = 2.53x − 0.422	0.04 ± 0.002	3.37 ± 0.12
ellagic acid (**60**)	301→284	0.9968	y = 8.93e−5x − 0.196	3.09 ± 0.25	7.97 ± 0.59
isorhamnetin 3-*O*-β-D-glucopyranoside (**61**)	477→301	0.9977	y = 5.1e−6x + 0.00474	10.61 ± 0.29	50.33 ± 1.87
quercetin-3-*O*-α-L-rhamnopyranoside (**63**)	447→301	0.9981	y = 0.00173x − 0.00746	---	3.06 ± 0.24
bartogenic acid (**96**)	517→455	0.9916	Y=0.000107x-0.154	3.59 ± 0.08	---

* Standard deviation of three independent experiments.

## Data Availability

The data presented in this study are available within the article and its supplementary material.

## Supplementary Materials

The following are available online at https://www.mdpi.com/2076-3921/10/2/278/s1, Table S1: LC–MS/MS conditions for quantitation of identified compounds by negative ion Multiple Reaction Monitoring (MRM).
