# Supplementary material for: Antioxidant and In Vitro Preliminary Anti-Inflammatory Activity of Castanea sativa (Italian Cultivar “Marrone di Roccadaspide” PGI) Burs, Leaves, and Chestnuts Extracts and Their Metabolite Profiles by LC-ESI/LTQOrbitrap/MS/MS
Source: Antioxidants (Basel). 2021 Feb 11;10(2):278. doi: 10.3390/antiox10020278 (PMC7917746; doi:10.3390/antiox10020278)
Supplement: Supplementary file 1 [file antioxidants-10-00278-s001.pdf]

antioxidants

Supplementary Materials: Antioxidant and *In Vitro* Preliminary Anti-Inflammatory Activity of *Castanea sativa* (Italian Cultivar "Marrone di Roccadaspide" PGI) Burs, Leaves, and Chestnuts Extracts and their Metabolite Profiles by LC-ESI/LTQOrbitrap/MS/MS

Antonietta Cerulli [1], Assunta Napolitano [1], Jan Hošek [2], Milena Masullo [1], Cosimo Pizza [1] and Sonia Piacente [1,*]

[1] Dipartimento di Farmacia, Università degli Studi di Salerno, via Giovanni Paolo II n. 132, I-84084 Fisciano (SA), Italy; acerulli@unisa.it (A.C.); anapoli@unisa.it (A.N.); mmasullo@unisa.it (M.M.); pizza@unisa.it (C.P.)
[2] Division of Biologically Active Complexes and Molecular Magnets, Regional Centre of Advanced Technologies and Materials, Faculty of Science, Palacký University, Šlechtitelů 27, 78371 Olomouc, Czech Republic; hosek.jan@vri.cz
* Correspondence: piacente@unisa.it (S.P.); Tel.: +39-089-969763; Fax: +39-089-969602

Table S1. LC–MS/MS conditions for quantitation of identified compounds by negative ion MRM.

Compound	DP	CE	CXP
Crenatin (**7**)	-37	-28	-19
chestanin (**21**)	-37	-44	-33
cretanin (**32**)	-54	-36	-24
quercetin 3-*O*-β-D-glucopyranoside (**54**)	-37	-30	-31
ellagic acid (**60**)	-37	-40	-31
isorhamnetin 3-*O*-β-D-glucopyranoside (**61**)	-21	-26	-23
quercetin-3-*O*-α-L-rhamnopyranoside (**63**)	-75	-34	-26
bartogenic acid (**96**)	-37	-44	-23

Declustering potential (DP), collision energies (CE), and collision cell exit potential (CXP)
